# Supplementary material for: The interactome of intact mitochondria by cross-linking mass spectrometry provides evidence for coexisting respiratory supercomplexes
Source: Mol Cell Proteomics. 2017 Dec 8;17(2):216–32. doi: 10.1074/mcp.RA117.000470 (PMC5795388; doi:10.1074/mcp.RA117.000470)
Supplement: Supplemental Data [file supp_RA117.000470_133922_0_supp_23978_fzffwf.zip › spectra_annotation/mito_spectra_annotation/331-1-4-1-9-1.pdf]

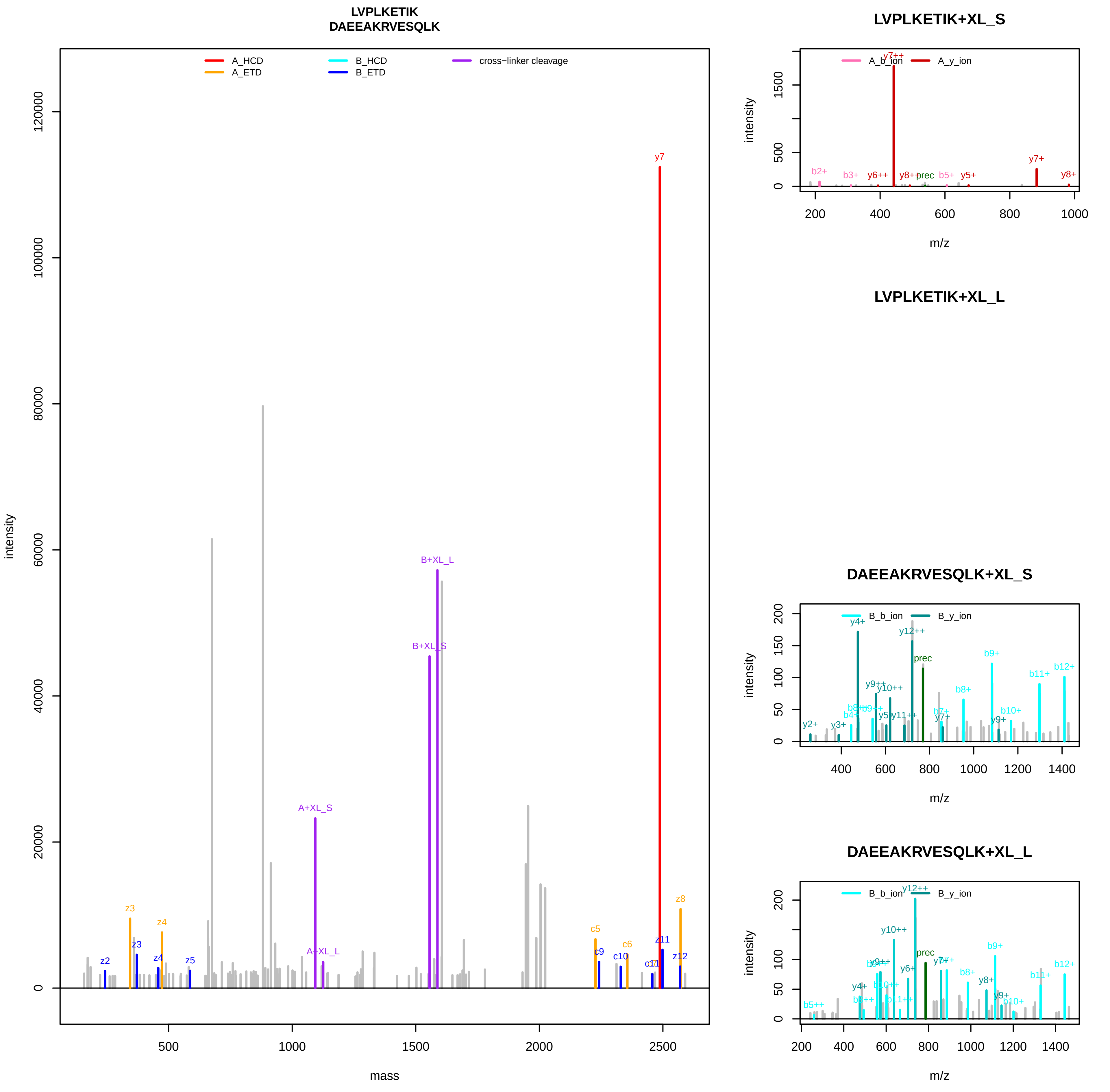
LVPLKETIK
DAEEAKRVESQLK
A_HCD
A_ETD
B_HCD
B_ETD
cross−linker cleavage
intensity
mass
y7
B+XL_L
B+XL_S
A+XL_S
A+XL_L
z2
z3
z4
z5
c5
c6
c9
c10
c11
z11
z12
z8
LVPLKETIK+XL_S
A_b_ion
A_y_ion
y7++
b2+
b3+
y6++
y8++
prec
b5+
y5+
y7+
y8+
m/z
LVPLKETIK+XL_L
DAEEAKRVESQLK+XL_S
B_b_ion
B_y_ion
y4+
y12++
b9+
b11+
b12+
b8+
y9++
y10++
y2+
y3+
y5+
y11++
b7+
y7+
b10+
y9+
DAEEAKRVESQLK+XL_L
b5++
y6+
y8+
